# Effect of combined and intensive rehabilitation on cognitive function in patients with Alzheimer’s disease evaluated through a randomized controlled trial

**DOI:** 10.1038/s41598-025-93236-6

**Published:** 2025-03-08

**Authors:** Michal Vostrý, Vlastimil Chytrý, Patrik Ch. Cmorej, Otakar Fleischmann, Nela Kubová

**Affiliations:** 1 Department of Occupational Therapy, Faculty of Health Studies, J. E. Purkyně University in Ústí Nad Labem, Ústí nad Labem, Czech Republic; 2 Department of Special and Social Pedagogy, Faculty of Education, J. E. Purkyně University in Ústí Nad Labem, Ústí nad Labem, Czech Republic; 3 Department of Pre-Primary and Primary Education, Faculty of Education, J. E. Purkyně University in Ústí Nad Labem, Ústí nad Labem, Czech Republic; 4 Department of Emergency Medicine, Radiology and Biomedical Technology, Faculty Health Studies, J. E. Purkyně University in Ústí Nad Labem, Ústí nad Labem, Czech Republic

**Keywords:** Alzheimer disease, Cognitive function, Special education intervention, Occupational therapy intervention, Combined therapy, randomized controlled trial, Quality of life for older adults, Environmental social sciences, Psychology and behaviour, Health care, Quality of life

## Abstract

This study investigates the impact of combined special education and occupational therapy intervention on cognitive functions in Alzheimer’s patients. Specifically, it evaluates changes measured by the Addenbrooke’s Cognitive Examination (ACE-R) after six months compared to a control group receiving standard care. A longitudinal, controlled experiment was conducted with random assignment to experimental and control groups. The experimental group underwent three weekly interventions of 45–50 min over eight months in 2021. Cognitive functions were periodically assessed using ACE-R. Power analysis determined a sample size of 128 participants for adequate statistical power; the study included 60 participants (30 per group). Data were analyzed using non-parametric methods due to non-normal data distribution. The experimental group showed significant improvement in ACE-R scores compared to the control group. The mean difference in scores was 10.27 points (SD = 2.83) for the experimental group, indicating improved cognitive function, while the control group showed a mean decrease of 5.67 points (SD = 2.06). Statistical analysis confirmed significant differences between groups at both interim and final assessments (p < 0.001). The combined special education and occupational therapy intervention led to significant cognitive improvements in Alzheimer’s patients compared to standard care. The study supports the efficacy of such interventions in enhancing cognitive functions, as evidenced by the substantial score increases in the experimental group.

## Introduction

Alzheimer’s disease is defined as a neurological, degenerative condition. As such, it gradually and significantly affects memory, executive functions, visuospatial abilities, attention, and eventually penetrates all other cognitive functions. Today, it is still considered the most common form of dementia among older adults. It is estimated that it accounts for about 60% of all diagnosed cases of dementia. Globally, the number of dementia cases is estimated to be around 36 million. Given the demographic trend of an aging population, for example, it is expected that by 2050, the number of cases will increase to approximately 115 million^1–3^. Additionally, various screening tools can be used to recommend individuals for more detailed examination. These tools include, for example, the Montreal Cognitive Assessment and the Mini-Mental State Examination (MMSE), which is the most well-known and commonly used short screening tool for assessing the overall level of cognitive impairment in clinical settings. However, recent studies suggest that for evaluating the overall functioning of older adults with cognitive impairment, it is appropriate to combine cognitive and functional tests, with both measures being understood as part of a single dimension of global functioning that can be assessed as a whole. Pharmacological treatments, such as donepezil, galantamine, or rivastigmine, have been shown to be insufficient or ineffective in patients with mild cognitive impairment. For this reason, it is necessary to focus on the development of non-pharmacological therapeutic approaches for this disease^4–6^. Cognitive stimulation is one of the most commonly recommended non-pharmacological approaches for alleviating cognitive problems in people with mild cognitive impairment (MCI) or mild to moderate dementia. Research in this area shows that cognitive stimulation and orientation therapy lead to an improvement in overall cognitive abilities and sometimes also to better quality of life and a sense of well-being, especially in individuals with milder stages of dementia. Moreover, recent studies suggest that cognitive stimulation therapy (CST) has a positive impact on improving general cognitive abilities, language skills, and quality of life in patients with mild to moderate dementia^7,8^. Recently, there has also been increasing interest in physical training (PT) as a tool for preventing dementia and slowing cognitive decline. This training includes endurance exercises and activities aimed at developing strength, balance, and flexibility. The goal of these exercises is not only to improve physical abilities such as strength and balance but also to support various cognitive functions, including attention, memory, and executive functions. Aerobic exercise has been shown to be particularly effective in improving overall cognitive abilities, with a smaller but significant effect on memory. Furthermore, regular aerobic activity promotes brain health by helping maintain neuronal balance and counteracting the aging of brain cells through protective and regenerative mechanisms. From a behavioral perspective, aerobic exercise improves mood and positively affects cognitive functions in various age groups, including spatial learning, attention, and executive functions. Additionally, some activities, such as physical training, which also stimulate cognitive activities, have been shown to bring significant benefits to brain health and overall well-being^9–11^. Under these circumstances, non-pharmacological interventions can be considered a complementary option for improving or maintaining cognitive functions, the ability to perform daily activities, or overall quality of life. This type of intervention, including cognitive training, stimulation, and rehabilitation, can bring significant benefits to older adults, both those with mild cognitive impairments and patients with dementia. Cognitive training involves tasks designed to strengthen specific cognitive abilities, such as memory, attention, or language skills, often combining home and supervised training. For example, this type of exercise may involve regular orientation in time and space using calendars or images with seasons, helping patients maintain temporal orientation^12–14^. Cognitive stimulation is usually provided through social activities or group discussions aimed at maintaining or improving both cognitive and social functions. On the other hand, cognitive rehabilitation focuses on practical skills in everyday life, such as learning or relearning important information, often involving family members or healthcare professionals in the process. In this way, older adults are offered the opportunity to maintain the best possible functioning based on an individualized approach^15,16^. Various systematic reviews have focused on the effectiveness of these cognitive interventions for people with dementia, but their results are often conflicting. Some studies suggest that cognitive training can have moderate to medium positive effects on overall cognitive abilities and verbal fluency, with these benefits persisting for several months after the intervention ends. On the contrary, other research has not found evidence that combined cognitive training and stimulation improve overall cognition in patients with dementia. However, cognitive stimulation has been associated with a slight improvement in cognition and quality of life in dementia patients, although the results may vary depending on the methods and setting used. It is important to note that improvements in cognitive functions, as measured by tests such as MMSE and ADAS-Cog, are not always clinically significant. According to one analysis that included data from multiple studies, cognitive training appears to be the most effective approach to improving cognitive functions in patients with Alzheimer’s disease compared to cognitive stimulation and rehabilitation. However, the relationship between the duration of these interventions and the persistence of their effects remains unclear and requires further investigation^17–19^.

## Methodology

The research study was designed as a longitudinal study comparing the experimental and control groups. Both groups regularly participated in social service activities. The experimental group, in addition to these activities, participated in interventions 3 times a week for 45–50 min. The interventions lasted for 6 months in 2021, with periodic testing. Initial testing took place in February 2023, followed by intensive interventions until May 2021, control testing in June, a break until August, and resumed intensive therapies until September 2021, concluding with final testing. The testing schedule was planned in accordance with the intervention timeline to reflect changes in cognitive functions during and after the intervention. The intervals between the testing phases were not strictly equal due to the nature of the intervention and organizational constraints. These timing specifics have now been added to the methodology section of the study. The interventions combined individual and group approaches to support cognitive functions and social adaptability. The research corresponds to a controlled experiment, with random assignment of participants to the experimental and control groups. Quota sampling of participants considered age, duration of diagnosis, length of stay in the facility, and region. Although it was not possible to fully meet the conditions of independence and homogeneity, these conditions were ensured as accurately as possible. The research design takes into account both probabilistic and non-probabilistic sampling types, with stratified random sampling used to divide the population into homogeneous units, from which the experimental and control groups were randomly selected.

### Power analysis

For the power analysis (conducted in Python), the following parameters needed to be defined: significance level (α): 0.05; power of the test (1–β): 0.80; effect size: using Cohen’s d, where we anticipated a medium effect size, i.e., (d = 0.5). Subsequently, we defined the use of a t-test for independent groups. The calculation showed that to achieve the desired statistical power (0.80) with a significance level (0.05) and the expected effect size (0.5), approximately n = 64 participants were needed in each group (experimental and control). Therefore, the total number of participants should be n = 128.

### Allocation concealment

We used several methods to achieve effective allocation concealment in our research, including central randomization and the use of concealed or sealed envelopes containing group allocation information. This ensured that those conducting the allocation were unable to know in advance which group a particular participant would be allocated to. The allocation of groups was therefore a random selection.

### Research sample

**Diagram 1:**
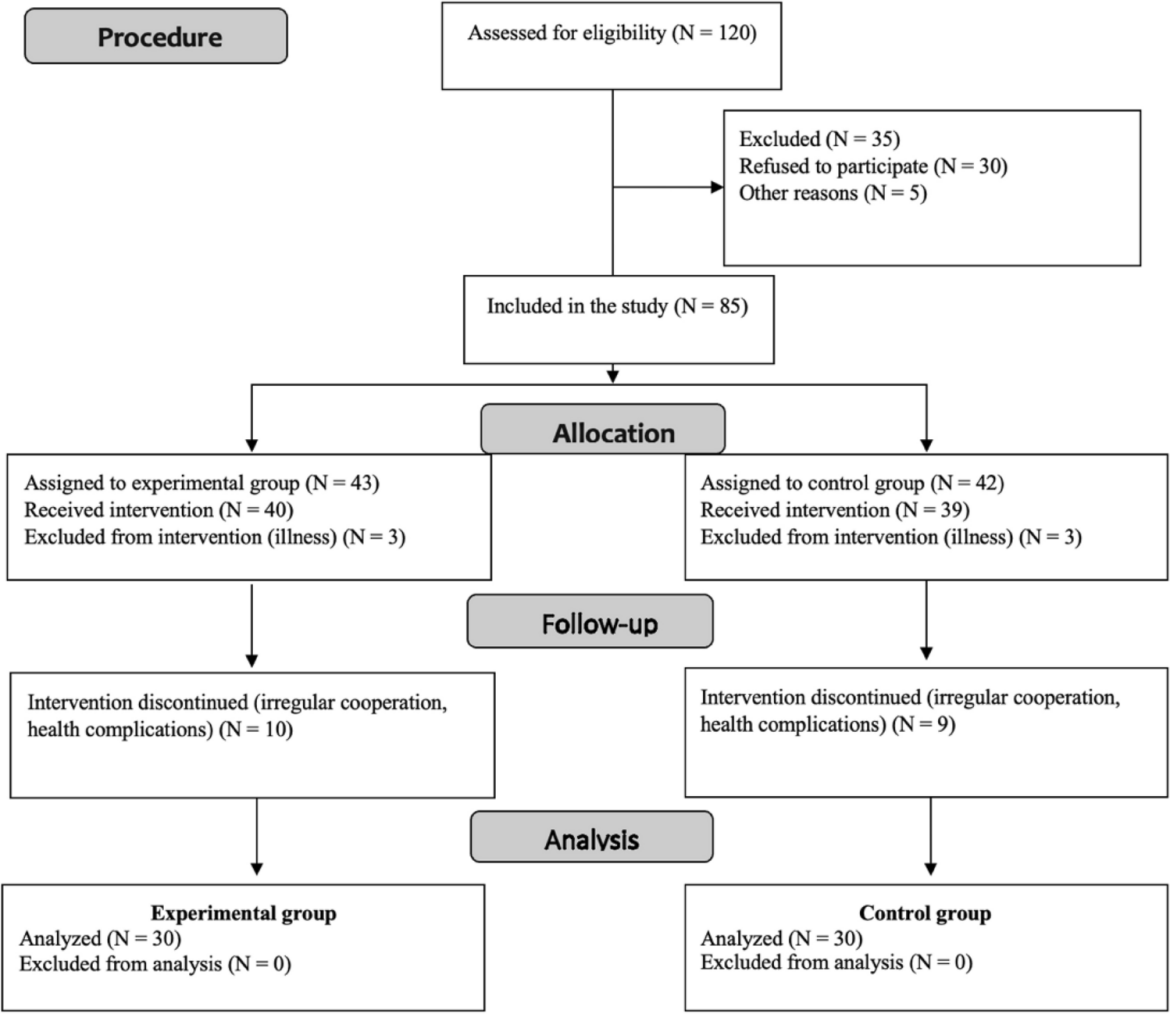
Flow Diagram Mapping the Step-by-Step Selection of the Research Sample into Experimental and Control Groups with the Final Number of Subjects Who Completed the Chosen Intervention Approaches.

Power analysis indicated that approximately 128 participants were needed, divided evenly into two groups. In our study, we worked with a sample of 60 participants (i.e., 30 participants in the experimental group and 30 participants in the control group). Based on the power analysis, we selected a total of 120 intentionally chosen participants who met the pre-defined relevant criteria (*at least 4 years since the diagnosis of Alzheimer’s disease dementia; according to ICD-10: Dementia in Alzheimer’s disease with late onset: F00.1; G30.1* + *; age over 65 years, placement in a nursing home for more than one year, and consent to participate in the intervention*). We then randomly divided this research sample into two groups. Eligibility criteria were established to ensure a homogeneous and comparable sample while minimizing confounding variables. The main inclusion criteria included age over 65, at least four years since the diagnosis of Alzheimer’s disease (ICD-10 criteria), placement in a care facility for over one year, and voluntary consent. These criteria were chosen to target a population where the intervention’s effects could be reliably assessed. Regarding exclusion criteria, we omitted participants with severe cognitive impairment (defined as scoring below 18 points on the MMSE) to ensure that the intervention would be applicable and beneficial within a mild-to-moderate dementia range. Additionally, participants with co-morbid conditions that could significantly affect cognitive function (e.g., advanced Parkinson’s disease, stroke, or severe psychiatric disorders) were excluded. This was done to reduce variability in outcomes that could stem from factors unrelated to Alzheimer’s disease itself. These inclusion and exclusion criteria were guided by findings from previous studies, which highlight the importance of targeting individuals in the earlier stages of dementia for interventions to have the most meaningful impact. A detailed description of the rationale behind these criteria has now been added to the methodology section for greater clarity. Diagram 1 shows the formation of the final research sample, which participated in our study for 6 months. The study began with 120 participants, who were selected using quota sampling and then randomly assigned to the experimental (43) and control group (42). Due to health complications, it was not possible to start the intervention with 6 participants, so the experimental group ultimately had 40 and the control group had 39 participants.

The control group was composed according to the same criteria as the experimental group: participants aged over 65, with at least four years since the diagnosis of Alzheimer’s disease (ICD-10), a minimum of one year in a care facility, and voluntary consent to participate. The control group did not undergo any interventions other than standard care provided by the facility. This information has been included in the methodology section.

The intervention was tailored to the health status, cognitive level, and specific needs of the participants, including the use of compensatory aids and adjustments to the intensity and duration of the intervention. Specific examples of these adaptations have been added to the text. Missing data were handled by including only participants who completed all phases of testing in the analysis. This approach has been detailed in the methodology section to ensure full transparency. The difference in sample size compared to the original power analysis (which recommended 128 participants) was due to organizational and financial constraints, resulting in a study with 60 participants (30 in each group). Nevertheless, we ensured a high statistical power (above 0.80), and this has been noted as one of the study’s limitations. The reasons for the difference in sample size and its potential impact on the results have now been explained in more detail in the text. The experimental group underwent regular and intensive therapy with testing before, during, and after the intervention. The control group was tested only at the same intervals. The final analysis included 30 participants from each group. Key anamnestic data are shown in Table 1. *All procedures conducted in this study adhered to the relevant guidelines and regulations as required by ethical approval. This includes ensuring the protection of the rights and dignity of all participants involved.*

**Table 1:** Characteristics of the research sample with respect to key anamnestic data.

Atributes	Experimental group	Control group
Age Ø (Average age)	69.2	70.2
Gender (%)		
Women	37 (93.0)	37 (95.0)
Men	3 (7.0)	2 (5.0)
Partnership status (%)		
Yes	17 (42.0)	20 (51.0)
No	23 (58.0)	19 (49.0)
Duration of illness in years ø (average Duration)	5,1	4,9
Education (%)		
University	7 (18.0)	4 (10.0)
Secondary school	20 (50.0)	16 (41.0)
Primary school	13 (32.0)	19 (49.0)
Duration Of Stay In The Facility Ø (Average Duration)	1,2	1,6
Medication (limiting; %)		
Yes	10 (25.0)	14 (36.0)
No	30 (75.0)	25 (64.0)
CO-Morbid diagnosis (limiting; %)		
Yes	5 (12.0)	8 (21.0)
No	35 (88.0)	31 (79.0)
Worsened food intake (%)		
Yes	3 (7.0)	1 (3.0)
No	37 (93.0)	38 (97.0)
Incontinence (%)		
Yes	15 (37.0)	19 (49.0)
No	25 (63.0)	20 (51.0)
Employment (%)		
Yes	30 (75.0)	28 (72.0)
Irregular	7 (18.0)	10 (26.0)
No	3 (7.0)	1 (4.0)
Family intervention (%)		
Yes	15 (38.0)	17 (44.0)
Irregular	20 (50.0)	20 (51.0)
No	5 (12.0)	2 (5.0)

### Method of data collection

From the perspective of assisting professions, we have excluded examinations that fall strictly within the domain of medical practice. These include laboratory tests, genetic testing, imaging methods, and electrophysiological investigative methods. A globally recognized scale/test is the Mini-Mental State Examination (MMSE). This test is primarily used to detect the presence and approximate severity of dementia. The MMSE is defined as a performance test that assesses cognitive functions through thirty questions. Evaluating the questionnaire is relatively easy and quick: one point is awarded for each correct answer, and zero points for incorrect answers, with a maximum score of 30 points. The approximate scoring breakdown is as follows:

30–27 points: normal cognitive function.

26–25 points: borderline findings, possibly indicating mild cognitive impairment.

24–18 points: indicative of mild dementia.

17–6 points: indicative of moderate dementia.

6 points or less: indicative of severe dementia

A more comprehensive version is the Addenbrooke’s Cognitive Examination (revised version from 2010). This test combines elements of both the MMSE and ACE-R into a single comprehensive test consisting of 18 tasks categorized into five domains: attention and orientation, memory, verbal fluency, language, and visuospatial abilities. The maximum score achievable on this test is 100 points. A cutoff score of 88 points is considered borderline, capable of detecting dementia, particularly Alzheimer’s disease. **Operationalization:** Cognitive function change is measured using the standardized ACE-R (Addenbrooke’s Cognitive Examination-Revised) test. The test comprises five core domains focusing on attention and orientation, memory, verbal fluency, language, and visuospatial abilities.

It is considered an extended version of the MMSE (Mini-Mental State Examination), allowing for a maximum score of 30 points on the MMSE and 100 points on the ACE-R test. Some tasks are overlapping between the two tests.

### Blinding

The implementation of blinding in our research involved several levels. We used single blinding, which means that only the participants were unaware of the group assignments, and double blinding, where both the participants and the researchers (or outcome assessors) were unaware of the group assignments. This approach ensured the highest possible level of objectivity and reduction of bias. In conclusion, blinding was a fundamental methodological practice in our research, which ensured higher validity, reliability, and ethical integrity of the study results. By preventing various forms of bias, we ensured that the study results accurately reflected the true effects of the intervention, thereby contributing to the advancement of scientific knowledge and the improvement of evidence-based practices.

### Intervention

To evaluate changes in cognitive functions, we utilized the standardized Addenbrooke’s Cognitive Examination – Revised version (ACE-R). This comprehensive test covers five domains: attention and orientation, memory, verbal fluency, language, and visuospatial abilities. The ACE-R builds upon the Mini-Mental State Examination (MMSE), offering a maximum score of 30 points for the MMSE and 100 points for the ACE-R. To assess functional independence, we employed the Barthel Index, which measures the level of dependency. Evaluations were conducted by trained assessors who were blinded to the study’s objectives. Our study underscores the innovative intervention strategies that integrate modern technologies and highlight the importance of regular and intensive interventions. Interventions were carried out with informed consent and tailored to the individual pace and needs of the participants. Key elements included the use of compensatory and special educational aids, modified according to the age and specific requirements of the participants. Emotional schemas, time visualization, and structural aids for daily and weekly routines were crucial in improving orientation and supporting cognitive functions. Cognitive training was enhanced with innovative technologies, including touch tablets, gaming consoles with virtual reality, and motion sensors, providing interactive and immersive therapeutic experiences. The use of 3D reality for fine motor skills and cognitive support demonstrates advanced therapeutic methods combining special education and occupational therapy approaches. The interventions encompassed comprehensive development of fine and gross motor skills, communication and social competencies, and cognitive functions. Methods such as psychomotor therapy, therapeutic physical education, individual and group discussions, and training in daily activities were implemented. Specific techniques included orientation in the environment, working with maps, route planning, and using daily schedules. Virtual reality was employed to support motor skills and cognitive functions through immersive and interactive exercises, minimizing the risk of dizziness. Relaxation after interventions was an important part of the therapy. The interventions were tailored to the individual needs of participants based on their health conditions, cognitive abilities, and medication use. This process included adjustments to the duration and intensity of activities, the use of specific compensatory aids (such as visual aids or adapted worksheets), and consideration of participants’ daily routines. Specific health issues, such as mobility limitations or difficulties with maintaining attention, were also taken into account. A more detailed description of this adaptation process has now been included in the text to facilitate potential replication of the study. The aim was to create a structured yet flexible daily routine that supports orientation and cognitive abilities while minimizing stereotypical activities. The control group participated in regular activities provided by the social services of the facilities where they were placed. These activities primarily included group sessions aimed at supporting social interaction, cognitive stimulation, and maintaining daily routines. The frequency of these activities was approximately 2 to 3 times per week, with each session lasting 45 to 60 min. Typical activities included social games, group discussions, working with photographs, and other activities designed to encourage group engagement.

### Statistical methods

To evaluate changes in cognitive functions, we used the standardized Addenbrooke’s Cognitive Examination – Revised version (ACE-R). This test encompasses five domains: attention and orientation, memory, verbal fluency, language, and visuospatial abilities. The ACE-R extends the Mini-Mental State Examination (MMSE), with a maximum score of 30 points for the MMSE and 100 points for the ACE-R. To assess functional independence, we used the Barthel Index, where the score indicates the level of dependency. The testing was conducted by trained evaluators who were unaware of the study’s aim. The preliminary study was conducted monthly in 2021, while the main study included three assessments: initial, control, and final.

Data were analyzed using Microsoft Excel 2016 and TIBCO Statistica 13, with the effect size calculated online. To compare changes in cognitive and functional abilities, we used the initial and final measurements from the ACE-R and Barthel Index. Normality of the data was tested using the Shapiro–Wilk test and further discussed with regard to sample size, data skewness, and the Q–Q plot. This issue is described in detail in the “Hypothesis Testing” chapter. R-M ANOVA and an unpaired t-test were used. Statistical significance was determined using p-values, with post-hoc analysis.

### Ethical aspects

All procedures adhered to the ethical standards of the national research committee and followed the 1964 Helsinki Declaration and its later amendments or comparable ethical standards. Our research team obtained administrative permission to access the data used in this study. We are aware that we are working with human subjects; therefore, the research study was approved by the Medical Ethics Committee of the Masaryk Hospital, prior to its commencement. All participants were properly informed about the nature of the research and provided their voluntary and informed consent. The intervention was designed to ensure that no physical or social harm would come to the participants, and their rights were fully respected. Registration number is 2021/12/12_001 (date of the firt registration 12.1.2024).

## Results

Table 2 presents the overall results of the Addenbrooke’s Cognitive Examination. In this test, it is possible to score a total of 100 points from various subdomains. The overall results indicate an improvement in scores for all participants in the experimental group with varying degrees of point gains. The highest score, 17 points, was achieved by one participant. Fifteen other participants improved their scores by more than 10 points. The remaining participants in this group had point gains ranging from 5 to 9 points, while the control group experienced a decline in scores by up to 9 points. This comparison clearly shows a positive impact on the experimental group. The presented results reflect the chosen intervention period during which our research study was conducted. Table 3 and Table 4 depict the results of descriptive statistics, Table 5 illustrates the difference between the experimental and control groups.

**Table 2:** Descriptive statistics of the experimental group for the Addenbrooke’s Cognitive Test – overall results.

Variable	Experimental group – Addenbrooke’s Cognitive Test, overall results					
	**Valid N**	**Ø**	**med**	**min**	**max**	**SD**
ADtot1	30	62,633	63,000	56,000	68,000	3,337
ADtot2	30	66,900	67,000	59,000	71,000	3,407
ADtot3	30	72,900	74,000	64,000	78,000	3,763

Legend: (ADtotP1 = overall results of the initial assessment; ADtotP2 = overall results of the control assessment; ADtotP3 = overall results of the final assessment).

**Table 3:** Descriptive statistics of the control group for the Addenbrooke’s Cognitive Test – overall results.

Variable	Control group – Addenbrooke’s cognitive test, overall results					
	**Valid N**	**Ø**	**med**	**min**	**max**	**SD**
ADtot1	30	63,133	63,000	56,000	68,000	2,862
ADtot2	30	61,033	61,500	56,000	66,000	2,710
ADtot3	30	57,467	57,500	53,000	62,000	2,556

Legend: (ADtotP1 = overall results of the initial assessment; ADto2 = overall results of the control assessment; ADto3 = overall results of the final assessment).

**Table 4:** Differences between experimental and control groups on the Addenbrooke’s Cognitive Test – overall results.

Variable	Differences in overall Addenbrooke’s Cognitive Test scores					
	**Valid N**	**Mean**	**Median**	**Minimum**	**Maximum**	**Std.Dev**
ADdif ─ experiment	30	10,267	10,000	5,000	17,000	2,828
ADdif ─ control	30	−5,667	−6,000	−9,000	−2,000	2,057

Legend: (ADdif – experimental = difference in results for the experimental group; ADdif – control = difference in results for the control group).

**Table 5:** Analysis of variance and practical significance for both groups in the Addenbrooke’s Cognitive Examination.

Contrast	Value p-level^a^	F statistic	Critical value	Cohen´s *d*	PE	Lower (CI)	Upper (CI)
Exp. in ─ Exp. cont	< 0,001	104,288	6,4558	2,28	−1,864	−2,455	−1,262
Exp. in─ Exp. out	< 0,001	395,494	6,4558	2,82	−3,631	−4,622	−2,633
Exp. con ─ Exp. out	< 0,001	265,4237	6,4558	2,017	−2,974	−3,811	−2,28
Con. in ─ Con. cont	< 0,001	36,6447	6,4558	1,900	1,105	0,643	1,556
Con. in ─ Con. out	< 0,001	227,7446	6,4558	2,057	2,755	1,959	3,541
Con. cont ─ Con. out	< 0,001	126,6772	6,4558	1,735	2,055	1,413	2,685
Exp. in ─ Kon. in	0,536	–-	–-	3,108	−0,161	−0,667	0,347
Exp. cont ─ Con. cont	< 0,001	–-	–-	3,078	1,906	1,287	2,513
Exp. out ─ Con. out	< 0,001	–-	–-	3,217	4,798	3,785	5,799

Legend: * = indicates a statistically significant difference; the commonly used evaluation of the coefficient d size is as follows ^20^: d ≥ 0.80 → large effect, d = 0.50–0.80 → medium effect, d = 0.20–0.50 → small effect; Exp. = experimental group, Con. = control group; in = input testing, cont = control testing, out = output testing;PE – point estimate; CI – 95% Confidence interval.

### Descriptive statistics

Based on the median values, it is evident that in the initial testing (ADtot1), the experimental and control groups were comparable (Med_exp_ = 63.00, Med_con_ = 63.00). The same conclusion would be reached using the mean values (Mean_exp_ = 63.133, Mean_con_ = 63.133). Differences between the two groups emerge only during the second (ADtot2) and third (ADtot3) testing.

### Hypothesis testing

Within the data analysis, the normality of the data was first evaluated (considering the sample size of N = 30 for both the experimental and control groups), also taking into account the possible assumption of the central limit theorem’s validity. The p-values from the Shapiro–Wilk test of normality were supplemented by skewness values: ADtot1exp (p = 0.27, skew = − 0.37); ADtot1con (p = 0.37, skew = − 0.37); ADtot2exp (p = 0.046, skew = − 0.60); ADtot2con (p = 0.179, skew = 0.18); ADtot3exp (p = 0.004, skew = − 1.10); ADtot3con (p = 0.14, skew = − 0.20). Some values (especially ADtot3exp) may indicate deviations from normality, as small samples are more sensitive to asymmetrical distributions. For subsequent analyses, parametric statistical methods will be used, primarily because these tests are appropriate due to their higher statistical power. In comparing three dependent datasets (ADtot1–3), R-M ANOVA was employed for both the experimental and control groups. Mauchly’s test of sphericity indicated that the assumption of sphericity can not be rejected, hence the assumption had not been violated, Exp—χ2(2) = 4.981, p = 0.083; Con—χ2(2) = 1.077, p = 0.584 (exp. group: Greenhouse–Geisser ε = 0.8599, Huynh–Feldt ε = 0.9089, Lower-bounde ε = 0.5; con. group: Greenhouse–Geisser ε = 0.9636, Huynh–Feldt ε = 1.0309, Lower-bounde ε = 0.5). The Repeated Measures ANOVA test indicated that there is a significant difference in the dependent variable between the different groups pro experimental group (F(2, 58) = 276.76, p < 0.001, with a mean of 62.63 for ADtot1, 66.9 for ADtot2, 72.9 for ADtot3) and for control group (F(2, 58) = 136.12, p < 0.001, with a mean of 63.13 for ADtot1, 61.03 for ADtot2, 57.47 for ADtot3). The observed effect size η2 is large for bots groups (η^2^_exp_ = 0.6; η^2^_con_ = 0.43) This indicates that the magnitude of the difference between the averages is large. The partial effect size is η_pexp_^2^ = 0,9052, η_pcon_^2^ = 0,8244. The test priori power is strong 0,9052 for experimenatl group and 0.9899 for control group.

From Table 5, it can be inferred that the experimental and control groups were comparable in the initial testing, both in terms of statistical significance and practical significance. The presented results of the cognitive test show a noticeable difference between the experimental and control groups in the final testing. For the difference in scores between the initial and final testing, the experimental group showed an increase in scores for all participants, with an increase ranging from 5 to 17 points (in one case). Conversely, all participants in the control group exhibited a decrease in scores, ranging from −3 to −9 points. A comparison of the resulting contrasts between the experimental and control groups was conducted, as well as between the initial, control, and final testing in terms of both statistical and practical significance. Our focus was primarily on the initial and control testing. The presented results indicate that there was no statistically significant difference between the initial testing and the scores in both groups. However, the difference in scores became apparent in the control testing, where a statistically significant difference was demonstrated (p-level < 0.001).

**Graph 1:**
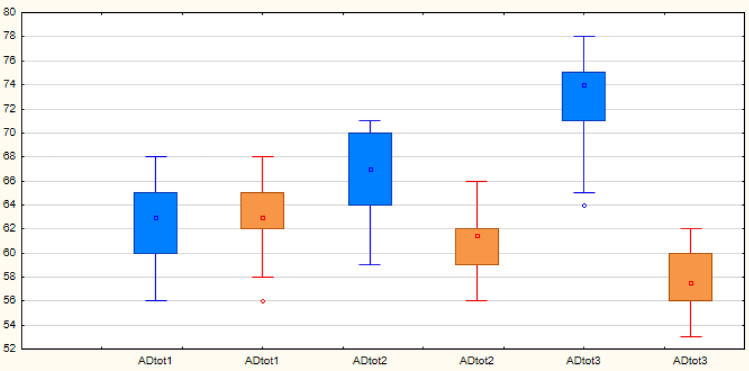
Representation of entry, control, and exit test scores in the experimental group (blue) and the control group (orange) for the Addenbrooke’s Cognitive Examination – overall results. Legend: ADtot1 – overall results of entry testing; ADtot2 – overall results of control testing; ADtot3 – overall results of exit testing; y-axis = score range of the subdomain in which the participants were situated.

This difference was also confirmed in the final testing (p-level < 0.001). We can therefore confirm that the experimental group showed a significant improvement in scores in the observed area compared to the control group. Significant differences are evident in all cases except for the initial assessment. These differences are also significant in terms of practical significance. Based on the presented results, we can accept the hypothesis that the synergy of special education and occupational therapy interventions will lead to better cognitive function outcomes in the experimental group after six months compared to the control group. Table 5 illustrates the analysis of variance and practical significance for both groups using the Addenbrooke’s Cognitive Examination. Graph 1 shows the score distribution for the initial, control, and final testing.

From the graph, it is evident that the experimental and control groups are comparable in the initial testing (ADtot1), where both the central values and the maximum values are exactly the same. The same applies to the minimum, although in that case it is due to an outlier in the control group. The third quartile is also the same. Differences can be observed in the interquartile range, where the control group has a narrower interval—this is attributable to a higher second quartile. When comparing all measurements, it is notable that in the control group, the interquartile ranges remain almost the same, shifting only downward along the vertical axis. In the experimental group, these ranges change in the third measurement, always shifting upward on the vertical axis.

## Discussion

The presented results obtained from our conducted research indicate improvements in the experimental group. Our goal was not to evaluate the quality of the provided services, but to assess our chosen intervention, which served beyond the usual activities offered to the participants in the respective facilities. By presenting the results, we have shown that if participants have a nearly daily regular and non-stereotypical program, their condition can be improved to some extent. It is important to recognize that the progression of the disease is relentless, and the more advanced the stage of the form of dementia (i.e., the longer the participants have been diagnosed), the less improvement we will see in terms of scores, with more stagnation and gradual loss of points in the observed indicators.

The primary recommendation we can formulate here is to target individuals who are in the early stages of dementia, meaning it has been diagnosed for up to three years. We base this on the fact that dementia lasts approximately nine years and ends in death. The early stage (mild form) lasts for three years, and this is where intensive comprehensive care is needed^21,22^. The crucial factor here is early detection of the disease. The earlier the condition is identified, the sooner the necessary cooperation with the patient can begin. Based on this cooperation, activities can be created to help prolong the quality of life of the senior. In the previous chapter, we presented the results of our research. The research focused primarily on the development of cognitive functions in the participants. Cognitive functions are weakened by the disease, leading gradually to immobility and permanent bed confinement with 24 care by others. The results of the research confirm our hypothesis that the created intervention combining special education and occupational therapy interventions would lead to improvements in the observed areas. As an additional standardized test, we used a test for managing daily activities. Here too, we demonstrated that participants showed slight improvements in these areas. By connecting these tests, we aimed to link the logical connection that better cognitive functions would also manifest in the management of daily activities, thus in social adaptability. The goal of our research was to determine whether the synergy of special education and occupational therapy interventions affects the level of cognitive functions in the tested participants of the experimental group compared to the participants in the control group. A total of 60 participants took part in the research throughout the intervention, selected by quota sampling. Based on the created research group, we randomly divided the participants into experimental and control groups, each with 30 participants. The experimental group participated not only in the activities of the facility but also in our chosen intervention. This intervention drew from the knowledge of special education and occupational therapy. The control group only participated in the activities of the facility. In the main study and subsequent data interpretation, we reached the following conclusions. Regarding the cognitive test, we demonstrated that our chosen intervention leads to better results in the observed areas compared to the control group, where the results either stagnated or worsened. It is important to mention that during the initial assessment, both the experimental and control groups scored at very similar levels, with no statistically significant difference between them (p^a^ = 0.647). This applies to the overall test evaluation, indicating that the initial cognitive test results were very similar for both groups. This finding was also supported by the quota sampling method we used, where we predefined relevant characteristics that all participants in our research group exhibited. The key comparison for us was evaluating the contrast between the initial and final testing for both groups and between the groups.

The presented results indicate the following findings: In the case of the cognitive test contrast in the experimental group, significant differences were demonstrated between the initial and final testing (p < 0.001), showing improvement in the observed areas of the test. For the control group, significant differences were also observed (p < 0.001), but the participants’ scores worsened in the observed areas of the cognitive test. This finding led us to accept our hypothesis. The presented results of descriptive and inferential statistics indicate, in most cases, a positive impact of our chosen intervention in favor of the experimental group. This can be simply interpreted to mean that our intervention automatically leads to improvement in the observed areas. However, from the analysis of professional sources, we encounter several controversies that highlight the still insufficiently understood impacts of both cognitive rehabilitation itself and innovative approaches on individuals diagnosed with dementia. We fully agree with this statement, and our chosen intervention aims to confirm the positive impact of these approaches, particularly cognitive rehabilitation, on seniors or other individuals with cognitive deficits. With this work, we primarily aim to enrich comprehensive rehabilitation through the cooperation of special education and occupational therapy, specifically targeting individuals diagnosed with a form of dementia or another cognitive deficit, especially in old age. The currently presented study and our previously conducted studies mostly indicate the positive impact of such approaches and the appropriate use of compensatory aids on development not only in the cognitive area but also in social adaptability. This raises the question of whether every such approach can inherently have an enriching character. We believe it can. Generally, if we provide intensive and long-term attention to these individuals, we can demonstrate certain improvements to a certain extent, despite the progressive nature of the disease. Unfortunately, in conducting our research, we encountered the reality of rather short-term and not very intensive or stimulating approaches. Based on these findings, we conducted this investigation to highlight the intriguing potential of combining these approaches.

Through this research, we aim to contribute to the general discourse surrounding the aging population and the increasing number of individuals with dementia. We hope to enrich the theoretical and practical realms in the helping professions and underscore the importance and effectiveness of comprehensive approaches for individuals suffering from dementia. In our selected research, we utilized a combination of several approaches that integrated elements of both special education and occupational therapy. In the application realm, we found many similar or related characteristics that these two fields share. In the application of modern technologies (hardware and software), we align with^23^. In his study, Bejan compares several information and communication technologies aimed at individuals diagnosed with dementia. His goal is to offer technologies that will help maintain the quality of life for individuals with disabilities in the near future.

Just as we have applied modern technologies, Bejan also points out certain reactions to be expected when working specifically with individuals with a form of dementia. Such reactions include initial verbal comments of curiosity. He also noted smiles and a positive attitude towards the use of these media in most participants. Bejan points out that the use of these technologies activated most participants both verbally and non-verbally. Let’s not forget the motor and cognitive stimulation provided by these approaches. Perceive modern technologies as an element of reminiscence therapy and emphasize their potential in the therapy of geriatric clients. The effectiveness of these technologies can thus intertwine with the relationship between the caregiver (interested person) and the client. Many authors highlight the positive impact of these approaches on both sides. They are often referred to as a “promising” way to innovate and develop implemented interventions. From a general perspective, such technologies can serve to improve decision-making confidence, reduce emotional tension, improve relationship conflicts, and increase self-efficacy^24^.

This applies to both the clients and the caregivers^25–27^. From the realization of our research, we also align with the conclusions of Starblanket et al. (2019), who point out the positive impacts of technologies on intervention approaches. However, they also highlight the insufficient use of these technologies in practice, or the lack of accessibility and inadequate equipment available in specific facilities. This makes it difficult to confirm the long-term impact of these approaches on potential improvements in various cognitive function components or on social adaptability and quality of life. Osvath et al. confirm the above. In their study, they state that these tools (modern technologies) offer unique opportunities to improve cognitive impairments. They approach the issue from a perspective very similar to ours in our study, emphasizing that using these tools facilitates daily life and can also reduce care costs. From a general perspective, technology helps maintain the abilities of the elderly and improve their daily functioning. In our study, we did not directly address the economic implications associated with hospitalization or long-term placement in a facility^28^. However, our main focus was on highlighting intervention approaches that lead to improvements not only in cognitive functions but also in social adaptability. We evaluated this in a modified manner within the relevant facility, considering several limitations. One significant limitation is that the elderly individual may be permanently placed in a nursing home or a similar facility that provides social services. This facility then becomes the senior’s everyday residence, where they not only move around and spend their leisure time but also establish social contacts. We can thus align with the author, as our chosen combined therapy led to improvements not only in cognitive but also in social skills, according to the results. However, in our case, we cannot attribute this outcome solely to modern technologies but to a combination of various approaches and their integration that we applied. At this point, we can state that modern technologies serve as a connecting link between special education and occupational therapy approaches. We perceive similar cooperation and blending of approaches in the application of cognitive rehabilitation. According to Carrion et al., it is a well-established intervention for treating seniors with dementia. It particularly highlights reality orientation and skill training, which seem to be effective interventions that can reverse cognitive impairment within a certain time frame, primarily in cases where the findings are not yet clear, or cognitive impairment is not extensive. In conclusion, the author mentions that skill training studies and mixed studies focusing on combining approaches can have a positive impact on cognitive functions. However, she emphasizes that it was difficult to better interpret the results in her study. The author compared several studies with different interventions and numbers of participants. Therefore, she concludes that more structured and comparable randomized controlled trials are needed. We also aimed to address this uncertainty mentioned by the author with our chosen intervention and conducted research, in which we also applied training of daily activities using various compensatory aids. This training led to a more positive adoption of these activities. As with modern technologies, regular supervision is necessary in this case as well^29^.

As known from special education and from individuals with intellectual disabilities, in our case, teaching an individual a specific activity does not mean they will automatically perform it.Both individuals with intellectual disabilities and those with Alzheimer’s type dementia gradually lose this skill and require long-term supervision.

This view is also supported^30,31^, who supplement our presented information by highlighting another positive impact, which they see in supporting the personality of these individuals and their regular use of cognitive skills. Analyses conducted by the authors indicate that such approaches are not only cost-effective but also beneficial. These approaches impact the overall quality of life, including the ability to manage daily activities.

They also point out the interesting fact that cognitive training can be applied in a home environment. By this, the authors primarily mean training family members or caregivers, who will use the same approaches and tools to communicate with the client. Although the authors do not explicitly name it as such, the article clearly indicates multidisciplinarity. Since there is currently no effective prophylaxis for dementia, much greater emphasis is placed on involving a large number of professionals, such as occupational therapists and psychologists. In this regard, we can also align ourselves^32,33^. The authors refer to this approach as non-pharmacological therapy, combining various approaches derived from the nature of the respective fields. Such approaches aim primarily to improve symptoms, reduce stress in both the client and the caregiver, enhance the living environment, and ultimately, as mentioned earlier, improve the quality of life.

The authors adeptly categorize non-pharmacological approaches into simpler ones, such as environmental interventions, and more complex ones, like the application of specific approaches (virtual reality, home automation etc.). However, we must realize that our goal is not to influence the underlying pathophysiological mechanisms but to maintain the function and participation of the client in the daily routine for as long as possible. Based on the above-presented information and our conducted research, we also wish to highlight the importance of the special educator as a vital member of the multidisciplinary team. Due to the nature of their profession, a special educator can contribute a range of specific intervention approaches and a modified approach that can be, to some extent, derived from psychopedagogical principles. Such a multidisciplinary team thus possesses a high-quality theoretical and practical foundation. To confirm the importance of the tests we have chosen, we refer, for example, to^34^, who in their study mention the main benefits of the Addenbrooke’s Cognitive Examination in our case. From their perspective, this test more thoroughly examines multiple cognitive functions.

The test itself better captures cognitive decline compared to the commonly used MMSE test, which is underscored by clinical experience. The application of the test is recommended for clients with mild impairment (mild cognitive impairment or early stages of dementia). The author also provides benchmark total score values, i.e., for dementia with the application of milder criteria, the test’s sensitivity is 94% and specificity is 89% (range or threshold). We are talking about an obtained score of 88 points. In the case of stricter criteria, the score is 82 points, with a sensitivity of 84% and specificity of 100%. This assertion was previously confirmed by Hummelová-Fanfrdlová et al. (2009), who estimate the test’s sensitivity at a score of 88 points to be 100%, and at a score of 83 points to be 96.6%. The same interpretation is shared by^35,36^.

Next authors present interesting results in their study, where they evaluated the outcomes of the Addenbrooke’s Cognitive Examination in 48 participants aged 50.0–59.12 years, 60.0–69.12 years, 70.0–79.12 years, and over 80 years. Additionally, participants were categorized based on the length of their education. According to the length of education, the results were as follows: for the group with 4–7 years of education, the descriptive data were mean = 80.25, SD = 7.58, Median = 80; for the group with 8–11 years of education, the results were mean = 84.75, SD = 6.95, Median = 84.5; for the group with more than 12 years of education, the results were mean = 27.21, SD = 1.38, Median = 27. The authors present normative data for these age groups concerning the length of education achieved. This study confirms the dependent relationship between cognitive abilities and demographic variables, such as age and level of education^37^. Next autothors in their study highlight the results of applying the Addenbrooke’s Cognitive Examination in a sample of 217 participants. The internal validity was assessed with a Cronbach’s alpha of 0.927, inter-rater reliability with an intraclass correlation coefficient of 0.976, and test–retest reliability with a kappa of 0.995. Based on these results, they validated the instrument for the Spanish population and consider it a reliable and valid test for diagnosing dementia^13^. The results of other studies are further confirmed by^38^, who in their study reached almost identical conclusions regarding the formulation of conclusions and recommendations for practice. Based on the conclusions of our research, we also align with these results^39^.

### Study limitation

**Despite the promising findings, our study has several limitations that should be acknowledged:** Sample Size: A power analysis showed that a sample size of approximately 128 participants is needed to achieve an adequate level of statistical power. Due to limited resources, the final sample size was nearly half of that recommendation, with 30 participants in each group. This reduction in sample size may lead to decreased statistical power of the tests, meaning a higher likelihood that some real effects will remain undetected (i.e., the risk of a Type II error). For this reason, the power of the test was calculated in each instance of using R-M ANOVA (commonly, according to Cohen (1988), values > 0.8 are considered sufficient). A value of 0.9899 means that the test has a 98.99% chance of detecting a difference between measurements, assuming such a difference truly exists. In this case, the probability of a Type II error is: β = 1—power = 1—0.9899 = 0.0101 = 1,01%. That means there is a very small chance the test would fail to detect a real effect.

**Duration of the Study:** Although the intervention lasted eight months, a longer follow-up period could provide more information about the long-term effects and sustainability of the observed improvements. The limited duration of the intervention and the ability to conduct planned retention tests were influenced by budgetary and time constraints, potentially impacting our ability to monitor long-term effects.

**Homogeneity of the Sample:** Efforts were made to ensure that both groups were equivalent in relevant characteristics before the intervention, although minimal differences were observed.

The participants were relatively homogeneous in terms of demographics and disease severity. Future studies should include a more diverse population to enhance the generalizability of the findings.

**Natural Progression of Disease:** Alzheimer’s disease inevitably changes the condition of patients over time, which could have influenced our results. The natural progression of the disease was a factor that we could not fully control.

**Measurement Tools**: The reliability and validity of the measurement tools used were carefully monitored, achieving an acceptable level of reliability. However, relying primarily on the Addenbrooke’s Cognitive Examination (ACE-R) as the main measure of cognitive function, while validated, may not provide a comprehensive evaluation of the intervention’s impact. Incorporating additional cognitive and functional assessments could enhance the robustness of the findings.

**Environmental Factors:** We aimed to minimize any environmental influence on the results by rotating intervention locations and respecting participants’ comfort preferences. Despite these efforts, environmental factors may still have had some impact.

**Cross-Over Design:** The study did not implement a cross-over design due to time constraints and limitations at the facility. Although a preliminary single-case experimental design indicated that the absence of our interventions led to a regression in cognitive function to baseline levels, a full cross-over study could provide more robust evidence of the intervention’s efficacy.

**Intervening Variables:** Limited budget and time constraints influenced the length of the intervention and the ability to conduct planned retention tests. These factors potentially had a significant impact on our ability to monitor the long-term effects of the intervention.

Overall, while these limitations affected the interpretation of our results, they were acknowledged and discussed in the presentation of our scientific findings. Addressing these constraints in future research will help to further validate and expand upon the promising outcomes observed in this study.

## Conclusion

Our study concludes that the combined intervention of special education and occupational therapy has a positive impact on cognitive functions in individuals with mild dementia. Our findings confirmed the hypothesis that long-term and systematic care leads to improvements in areas affected by the progressive disease. The modern technologies and methodologies we applied demonstrated their potential in supporting daily activities and cognitive rehabilitation. We recommend continuing similar multidisciplinary approaches for further research and practical application, with the aim of improving the quality of life for individuals with dementia and their caregivers. The practical impact of our results is reflected in several key points:

**Improvement in Quality of Life for Dementia Patients:** Our study showed that the combined intervention of special education and occupational therapy has the potential to enhance daily functionality and social adaptability in individuals with mild dementia. This practical impact can lead to greater independence and an overall improvement in the standard of living for patients.

**Support for Caregivers:** The results suggest that a multidisciplinary approach can reduce the burden on caregivers. Through specific methods and techniques of occupational therapy, caregivers can be provided with tools to manage daily tasks and communicate with dementia patients more effectively.

**Innovation in Non-Pharmacological Intervention Practices:** The modern technologies and methodologies used in our study offer new perspectives for the treatment of dementia. Integrating these approaches into clinical practice could lead to more effective symptom management and enhanced overall care for patients.

**Further Research and Application:** Our results support continued research in the area of multidisciplinary approaches to dementia. Identifying specific methods that are most effective for different types of dementia and their stages can lead to broader application of these interventions and improvements in care standards.

## Data Availability

The datasets used and/or analysed during the current study available from the corresponding author on reasonable request.

## References

[1] Herrup, K. Commentary on recommendations from the national institute on aging Alzheimer’ s association workgroups on diagnostic guidelines for Alzheimer’ s disease Addressing the challenge of Alzheimer s disease in the 21st century. *Alzheimers Dement.* **7**, 335 (2011). 21575876 10.1016/j.jalz.2011.04.002

[2] World Health Organization. (2012). Dementia: A Public Health Priority. *World Health Organization,* 2012.

[3] Wang, Y. Y. et al. The effect of cognitive intervention on cognitive function in older adults with Alzheimer’s disease: A systematic review and meta-analysis. *Neuropsychol. Rev.* **32**(2), 247–273 (2022). 33893905 10.1007/s11065-021-09486-4

[4] Nasreddine, Z. S. et al. The Montreal Cognitive Assessment, MoCA: a brief screening tool for mild cognitive impairment. *J. Am. Geriatrics Soc.* **53**(4), 695–699 (2005). 10.1111/j.1532-5415.2005.53221.x 15817019

[5] Petersen, R. C. et al. Practice guideline update summary: Mild cognitive impairment: report of the guideline development, dissemination, and implementation Subcommittee of the American Academy of Neurology. *Neurology* **90**(3), 126 (2018). 29282327 10.1212/WNL.0000000000004826 PMC5772157

[6] Matías-Guiu, J. Validación de la versión española del test Addenbrooke’s cognitive examination III para el diagnóstico de demencia. *Neurologia* **30**(9), 545–551 (2015). 25002342 10.1016/j.nrl.2014.05.004

[7] Lauenroth, A., Ioannidis, A. E. & Teichmann, B. Influence of combined physical and cognitive training on cognition: A systematic review. *BMC Geriatrics* **16**, 1–14 (2016). 27431673 10.1186/s12877-016-0315-1 PMC4950255

[8] Lobbia, A., Carbone, E., Faggian, S., Gardini, S., Piras, F., Spector, A., & Borella, E. The efficacy of cognitive stimulation therapy (CST) for people with mild-to-moderate dementia. *European Psychologist* **24**(3), 257-277 (2019).

[9] Devenney, K. E., Sanders, M. L., Lawlor, B., Olde Rikkert, M. G. & Schneider, S. The effects of an extensive exercise programme on the progression of Mild Cognitive Impairment (MCI): study protocol for a randomised controlled trial. *BMC Geriatrics* **17**, 1–10 (2017). 28330458 10.1186/s12877-017-0457-9 PMC5361785

[10] Gillis, C., Mirzaei, F., Potashman, M., Ikram, M. A. & Maserejian, N. The incidence of mild cognitive impairment: A systematic review and data synthesis. *Alzheimer’s Dementia: Diagnosis, Assess. Dis. Monit.* **11**, 248–256 (2019). 10.1016/j.dadm.2019.01.004 PMC6416157 30911599

[11] Kiper, P. et al. Combined motor and cognitive rehabilitation: the impact on motor performance in patients with mild cognitive impairment Systematic review and meta-analysis. *J. Personal. Med.* **12**(2), 276 (2022). 10.3390/jpm12020276 PMC8874573 35207764

[12] Chiu, H. L. et al. The effect of cognitive-based training for the healthy older people: a meta-analysis of randomized controlled trials. *PLoS One* **12**(5), e0176742 (2017). 28459873 10.1371/journal.pone.0176742 PMC5411084

[13] Matias-Guiu, J. A. et al. Cognitive interventions for cognitivelyhealthy, mildly impaired, and mixed samples of older adults: A systematic review and meta-analysis of Randomized-Controlled trials. *Neuropsychol Rev.* **27**(4), 403–439 (2017). 28726168 10.1007/s11065-017-9350-8

[14] Smart, C. M. et al. Non-Pharmacologic interventions for older adults with subjective cognitive decline: Systematic review, Meta-Analysis, and preliminary recommendations. *Neuropsychol. Rev.* **27**(3), 245–257 (2017). 28271346 10.1007/s11065-017-9342-8

[15] Sherman, D. S., Mauser, J., Nuno, M. & Sherzai, D. The efficacy of cognitive intervention in Mild Cognitive Impairment (MCI): A Meta-analysis of outcomes on Neuropsychological Measures. *Neuropsychol. Rev.* **27**(4), 440–484 (2017). 29282641 10.1007/s11065-017-9363-3 PMC5754430

[16] Clare, L., Woods, R. T., Moniz Cook, E. D., Orrell, M. & Spector, A. Cognitive rehabilitation and cognitive training for early-stage Alzheimer’ s disease and vascular dementia. *Cochrane Database Syst. Rev* **4**, CD003260 (2003). 10.1002/14651858.CD003260 14583963

[17] Huntley, J. D., Gould, R. L., Liu, K., Smith, M. & Howard, R. J. Do Cognitive Interventions Improve General Cognition in Dementia?. *A Meta-Analysis and Meta-Regression. BMJ Open* **5**(4), e005247 (2015). 25838501 10.1136/bmjopen-2014-005247 PMC4390716

[18] Liang, J. H. et al. Comparison of Cognitive Intervention Strategies for Older Adults With Mild to Moderate Alzheimer’ s Disease: A Bayesian Meta-analytic Review. *J. Am. Med. Dir. Association* **20**(3), 347–355 (2019). 10.1016/j.jamda.2018.09.017 30459116

[19] Quan, W., Liu, S., Cao, M. & Zhao, J. A Comprehensive Review of Virtual Reality Technology for Cognitive Rehabilitation in Patients with Neurological Conditions. *Appl. Sci.* **14**(14), 6285 (2024).

[20] Cohen, J. Statistical Power Analysis for the Behavioral Sciences. *New York Routletge*. (1988).

[21] Pidrman, V. Demence-1 část: Diagnostika a diferenciální diagnostika. *Medicína pro praxi.* **4**(2), 83–88 (2007).

[22] Vostrý, M., Fischer, S. & Lanková, B. The Effect of combined therapy on the support and development of social skills of people with multiple sclerosis in senior age. *Neuroendocrinol. Lett.* **41**(5), 101–105 (2020). 33315341

[23] Bejan, A. et al. Developing a technology ‘wish-list’ to enhance the quality of life of people with dementia. *Gerontechnology* **6**(1), 2–19. 10.4017/gt.2007.06.01.002.00 (2007).

[24] Sixsmith, A. & Gibson, G. Music and the wellbeing of people with dementia. *Ageing Soc.* **27**(1), 127–145. 10.1017/S0144686X06005228 (2007).

[25] Gagnon, M. P. et al. Interventions for promoting information and communication technologies adoption in healthcare professionals. *Cochrane Database Syst. Rev.* **21** (1),1-34. 10.1002/14651858.CD006093.pub2 (2009). 19160265 10.1002/14651858.CD006093.pub2 PMC3973635

[26] Lindberg, B., Nilsson, C., Zotterman, D., Söderberg, S. & Skär, L. Using information and communication technology in home care for communication between patients, family members, and healthcare professionals: a systematic review. *Int. J. Telemed. Appl.*10.1155/2013/461829 (2013). 23690763 10.1155/2013/461829 PMC3649237

[27] Lucero, R. J. et al. The effects of information and communication technologies on informal caregivers of persons living with dementia: a systematic review. *Alzheimer’s Dementia: Transl. Res. Clini. Interv.* **5**, 1–12. 10.1016/j.trci.2018.11.003 (2019). 10.1016/j.trci.2018.11.003 PMC6315277 30623020

[28] Osvath, P. et al. The use of information and communication technology in elderly and patients with dementia. *J. Gerontol Geriatr Res.* **7**(475), 2. 10.4172/2167-7182.1000475 (2018).

[29] Carrion, C., Folkvord, F., Anastasiadou, D. & Aymerich, M. Cognitive therapy for dementia patients: a systematic review. *Dementia Geriatric Cognitive Dis.* **46**(1–2), 1–26. 10.1159/000490851 (2018). 10.1159/000490851 30092585

[30] Aguirre, E. et al. Cognitive stimulation therapy (CST) for people with dementia—who benefits most?. *Int. J. Geriatric Psychiatry* **28**(3), 284–290. 10.1002/gps.3823 (2013). 10.1002/gps.3823 22573599

[31] Gibbor, L., Yates, L., Volkmer, A. & Spector, A. Cognitive stimulation therapy (CST) for dementia: a systematic review of qualitative research. *Aging Mental Health* **25**(6), 980–990. 10.1080/13607863.2020.1746741 (2021). 32252561 10.1080/13607863.2020.1746741

[32] Bartolo, M. et al. Clinical scales for measuring stroke rehabilitation promote functional recovery by supporting teamwork. *Eur. J. Phys. Rehabilitat. Med.* **52**(2), 195–202 (2015). 25573601

[33] Poulos, C. J. et al. A comprehensive approach to reablement in dementia. *Alzheimer’s Dementia: Transl. Res. Clini. Interv.* **3**(3), 450–458. 10.1016/j.trci.2017.06.005 (2017). 10.1016/j.trci.2017.06.005 PMC5654482 29067351

[34] Bartoš, A., Raisová, M. & Kopeček, M. Novelizace české verze Addenbrookského kognitivního testu (ACE-CZ). *Česká a slovenská Neurologie A Neurochirurgie* **74**(6), 681–684 (2011).

[35] Nikolai, T., Vyhnálek, M., Literáková, E., Marková, H. & Hort, J. Vyšetření kognitivních funkcí v časné diagnostice Alzheimerovy nemoci. *Neurologie Pro Praxi* **14**(6), 297–301 (2013).

[36] Mioshi, E., Dawson, K., Mitchell, J., Arnold, R. & Hodges, J. R. The Addenbrooke’s Cognitive Examination Revised (ACE-R): a brief cognitive test battery for dementia screening. *Int. J. Geriatric Psychiatry: A J. Psychiatry late life Allied Sci.* **21**(11), 1078–1085. 10.1002/gps.1610 (2006). 10.1002/gps.1610 16977673

[37] Amaral-Carvalho, V. & Caramelli, P. Normative data for healthy middle-aged and elderly performance on the Addenbrooke Cognitive Examination-Revised. *Cognitive. Behav. Neurol.* **25**(2), 72–76. 10.1097/WNN.0b013e318259594b (2012). 10.1097/WNN.0b013e318259594b 22596112

[38] Kourtesis, P., Margioti, E., Demenega, C., Christidi, F. & Abrahams, S. Addenbrooke Cognitive Examination-III and detection of Alzheimer’s disease: a comparison of ACE-III, M-ACE and ECAS in a Greek Alzheimer’s disease population. *J. Int. Neuropsychol. Soc.* **26**(8), 825–834. 10.1017/S1355617720000314 (2020). 32312343 10.1017/S1355617720000314

[39] Palumbo, R. et al. Measuring global functioning in older adults with cognitive impairments using the Rasch model. *BMC Geriatrics* **20**, 1–14 (2020). 10.1186/s12877-020-01886-0 PMC7685614 33228541

